# The Attitudinal Style and Its Potential for Hybridization with Other Pedagogical Models: A Narrative Review

**DOI:** 10.3390/bs14030152

**Published:** 2024-02-21

**Authors:** José Luis Álvarez-Sánchez, Ángel Pérez-Pueyo, David Hortigüela-Alcalá

**Affiliations:** 1 Faculty of Physical Activity and Sport Sciences, University of León, 24004 León, Spain; 2 Faculty of Education, University of Burgos, 09001 Burgos, Spain

**Keywords:** attitudinal style, pedagogical models, hybridization, physical education

## Abstract

The ‘Attitudinal Style’ (AS) enhances the democratic and engaging learning process by improving student motivation and attitudes. Its adaptability, transferability, and applicability make it suitable for hybridization with other pedagogical models (PMs). This study explores the possibilities of blending AS with other PMs for classroom applications, emphasizing the use of formative and shared assessments to maximize efficiency. Adopting a Narrative Review methodology, the research delves into ten academic databases, identifying seven publications that meet the inclusion criteria for a detailed analysis. These publications propose pedagogical approaches for sports, body expression, natural environments, and physical conditioning. They highlight the potential of integrating AS with other PMs to not only enrich physical education (PE) experiences but also introduce innovative teaching methods for various physical activities. The evidence from these sources suggests that combining AS with other PMs serves as a robust strategy to enhance the overall educational experience in PE.

## 1. Introduction

In the realm of emerging pedagogical models (PMs), the Attitudinal Style (AS) stands out as an emerging PM that prioritizes fostering a positive attitude in learners, irrespective of their physical abilities. It aims to create successful experiences for all students, avoiding exclusion. This model focuses on creating positive experiences where movement serves as a means, rather than the end, of the learning process, consisting of three fundamental elements: (1) intentional body activities, (2) sequential organization toward attitudes, and (3) final assemblies [1]. Its interdisciplinary potential, applicability, and emphasis on fostering positive student attitudes make it an attractive choice for convergence with PMs.

It is crucial to highlight the AS’s strong global focus, making it applicable to various content areas. This facilitates the development of interdisciplinary proposals and ensures continuity, allowing for the verification of evident and measurable results in the long term. Like other emerging models, the AS presents an opportunity to revitalize PE instruction by generating concrete evidence of learning and accomplishment [2].

The AS approach goes beyond physical aptitude and motor skills, offering students unique opportunities to develop their cognitive and affective capacities through interpersonal relationships, social insertion, and a deeper understanding of PE’s value in their lives. However, implementing a single PM at the elementary or secondary levels has proven challenging due to the demands for sustained effort, a high level of pedagogical content knowledge, and increased involvement from both teachers and students [3]. Recognizing the limitations of a one-size-fits-all approach, some authors, such as [4,5], suggest that no single model can effectively promote learning across all diverse PE contexts. This realization led [6] to propose a multi-model approach, with [7] advocating for the hybridization of PMs to achieve this goal.

This growing trend in PM hybridizations suggests that this pedagogical strategy extends the impact of single PM implementations. Combining PMs based on common features enables teachers to introduce diverse content areas in PE, overcoming the constraints of individual models [8]. In essence, hybridizing PMs involves extracting and integrating essential features from two models or using one model as a base and adding critical components from another model [9,10]. Successful hybridizations of various models have been witnessed in different PE contexts, such as Sport Education (SE) and Teaching Games for Understanding (TGfU) [11,12]; Cooperative Learning (CL) and Teaching Personal and Social Responsibility (TPSR) [13]; TPSR and TGfU [14,15]; or SE and TPSR [16,17]. These hybridizations appear to enhance children’s and adolescents’ learning across the motor, cognitive, affective, and social domains, improving game performance, motor abilities, and psychological outcomes [18].

A notable aspect of the AS, beyond its efficiency and applicability, is its ease of hybridization with other PMs, along with the use of a formative and shared assessment as a key element of the model itself [1]. This plays an essential role in generating competency-based learning. While various practical and informative proposals for the model exist in the literature, there is no publication examining the state of the art or synthesizing all publications delving into the possibility of hybridizing the model. The main objective of the present research is to explore the state of the art, analyzing the possibilities of hybridization of the AS with different PMs with which it shares one or more characteristics. The specific objective is to determine the key elements that are needed to carry out the hybridizations and analyze the impact of these hybridizations on the generation of competency-based learning.

## 2. Methodology

This Narrative Review explores the hybridization of AS with other PMs in the context of PE. Its objective is to synthesize the existing research that delves into the integration of AS with various educational models to enhance the teaching and learning processes in PE. This review places particular emphasis on the diversity of content, the sports context, and the specificity of classroom applications.

The literature search, initiated on 9 September 2023, encompassed ten electronic databases recognized for their relevance across multiple disciplines that were aligned with our research focus. These databases included ERIC for education, Psicodoc and PsychInfo for psychology, SportDiscus for sports sciences, Dialnet and Scielo for Spanish-language scientific literature, and Scopus, PubMed, WOS Core Collection, and ProQuest Dissertation for multidisciplinary research. This comprehensive search strategy was employed to compile an exhaustive collection of the literature discussing the hybridization of AS within PE contexts.

The inclusion/exclusion criteria for publication selection were as follows: the publication needed to primarily address practical hybridizations within the context of PE while considering AS. Consequently, publications with theoretical aspects, and those exhibiting significant similarity (where identical data were reported in multiple publications, such as a journal article and electronic report), were excluded. Only publications with the most comprehensive datasets were considered. Researchers focused on selecting those most representative of content typically included in the PE curriculum, namely the natural environment, physical condition, sports, and body expression.

## 3. Results

### 3.1. Search Results

From an initial pool of 98 identified publications, the selection process refined the included publications to 7 key publications. This refinement involved the removal of duplicates and a rigorous screening for relevance based on the titles, abstracts, and keywords, applying eligibility criteria. Specifically, it was centered on the topic of practical AS hybridization in the PE context. Publications with insufficient practical detail in their content were excluded. During this process, with each record was independently reviewed by two researchers. Any discrepancies or disagreements were resolved through consultation with a third reviewer, striving for a unanimous consensus.

The publications were chosen because they addressed the diversity of content related to PE, the specificity of sports contexts, and direct applicability in classroom settings. This selection criterion ensured that the chosen studies provided a comprehensive understanding of how AS and other PMs can be integrated.

### 3.2. Studies Characterization

The studies included in this narrative review were carried out between 2011 and 2023. All the research was conducted in Spain and is of an informative nature. 

The general characteristics of papers included in this study are reported in Table 1.

## 4. Pedagogical Models for Hybridization

### 4.1. Teaching Games for Understanding and Attitudinal Style

The TGfU model emerged as a response to the traditional approach to teaching games in secondary schools, which mainly focused on techniques [26]. This model aims to help students understand both the technical and tactical aspects of sports by using modified versions of actual gameplay, like simplified or small-sided games [27,28]. These modifications include changes to elements such as space, the number of players, the rules, balls, and the goal, using principles like representation, exaggeration, and tactical complexity. By tailoring the sport to students through these modifications, the goal is to encourage more active participation in the game [29]. These pedagogical intentions are also framed within the context of AS, which involves introducing modifications to game situations and understanding the essence of sports before delving into the technique. In fact, in AS, the goal is to develop a methodology based on attitudes that can equally apply to all students in a group, regardless of their individual characteristics.

In TGfU, the focus is on guiding students to analyze diverse game situations, fostering problem-solving in a dynamic game environment. Student involvement in the learning process is considered a fundamental aspect of this teaching approach [27,30]. Teachers use questioning feedback on tactical principles to deepen students’ understanding of the game. Strategic questioning prompts active student engagement in the inquiry process [12]. Aligned with these principles, the TGfU methodology allows the integration of AS-perspective proposals at various unit phases. AS advocates for the application of a formative and shared assessment, aiming to generate the highest quantity and quality of learning. In this approach, evaluation is integrated into the entire teaching and learning process. From the beginning of the unit in the AS approach, students play a significant role. They actively observe, analyze, and organize themselves and their peers, make decisions, and continuously evaluate. For example, after the initial learning and group cohesion sessions, AS suggests assigning one group the role of observers in group tactical situations. This group, alongside the teacher, analyzes the game, identifies common mistakes, and assesses whether all peers participate in a balanced and equitable manner.

This participatory observation aligns with the TGfU approach, prioritizing cognitive learning before psychomotor skills’ development, emphasizing a holistic and participatory learning experience.

Additionally, concerning the final assemblies, and to maintain coherence in the activities throughout the unit, the AS suggests the group execution of two plays incorporating technical–tactical elements. These plays can be repeated several times, with each group member taking on different roles. This approach ensures that everyone, without exception, feels important within the group by contributing to a collective achievement [23]. During the actual performance, other groups will represent the play (scouting), providing an opportunity to assess students’ learning of the technical–tactical dimensions covered in previous classes. Integrating these elements alongside the TGfU model allows for a holistic and inclusive approach, fostering a positive learning environment for all students, including those who initially had lower self-esteem in team sports. 

This approach shifts the focus from the proficiency of the most skilled students to their involvement in the overall teaching process. This contributes to enhanced self-esteem and perceived competence among all students. In the realm of team sports, blending TGfU and AS ensures that each student, irrespective of their initial skill level, plays a vital role in the learning experience (both individually and collectively). This not only promotes a positive and supportive learning environment but also contributes to the holistic development of each student. In addition, it fosters a sense of accomplishment and competence within the context of PE, key aspects in both MPs.

### 4.2. Cooperative Learning and Attitudinal Style

CL is an educational methodology where small groups of students, each with diverse ability levels, collaborate on a shared task to enhance individual and peer learning. In this collaborative process, students actively contribute efforts and share resources [31,32,33]. This approach transcends mere group work, as students are not only motivated to master specific content but also strive to assist their classmates in reaching their own learning objectives [34,35]. This mirrors the approach proposed by AS, which starts with small groups, evolves into larger groups, and eventually becomes class-wide groups. Both models underscore the importance of socialization and the assumption of social responsibilities within a group. It is worth noting that the similarities shared by both approaches allow for formative evaluation processes and the evaluation instruments of AS to be used in CL.

However, although the AS has a strong cooperative component, it cannot be strictly classified as such because it incorporates a series of distinctive variables [1]. Among these variables, the initial effort of the teacher to promote the values, attitudes, and individual achievement of all students is highlighted. Furthermore, it seeks the development of the different capacities defined by [36], beyond motor capacities, but using these as the basis of integral development. The formation of initial groups is based on an initial affinity criterion, sequentially evolving towards attitudes. Then, it progresses to larger groups and culminates, in most cases, in a group challenge involving the entire class.

In the literature, various proposals are available in which a hybridization occurs between CL and AS. This involves selecting the strengths and advantages of each approach to adapt one approach to the specific context and content. For instance, the following examples stand out:

(1) Country dance [21]: this proposal begins with the teacher’s direct teaching of dance steps and simple choreographies that are repeated with different songs to enhance student motivation. The aim is to convey that everyone, without exception, can dance well. Following this initial phase, designed to prevent motor discrimination, the approach transitions to small group work under a cooperative framework. Students are given the responsibility to manage their progress [37]. The teacher emphasizes that the choreography will not be valid until all members of the group have learned it. Once they finish, following the sequential organization of attitudes, they will join with another group, and then with the whole class, to create a final assembly.
(2) Ropework [25]: In the initial phase, students learn three basic knots, emphasizing that the goal is for everyone to achieve proficiency. The teacher provides instructional materials through digital media, employing the flipped classroom methodology. Subsequently, in the classroom setting, students form groups based on an affinity criterion, aligning with the characteristics of AS regarding the creation of base groups. In this phase, the cooperative learning strategy “Aronson’s Puzzle” is applied. After learning the knots together, each group will select an expert from each knot, so expert groups are formed. Later on, and after a learning phase, each of the experts returns to their base group to explain the knot to their classmates, making sure that everyone learns it. The goal is for everyone to learn all the knots, developing positive interdependence, a characteristic of CL [38]. Finally, a proposal is generated in a large group, who can propose different activities that can be carried out using the previously created monkey bridges.
(3) Cooperative physical challenges with an emotional aspect [24], although they share certain characteristics with cooperative challenges [39], focus on the emotional–affective dimension rather than the intellectual–cognitive one. That is why they aim for all students to experience all roles, especially the role of the agile/performing person. In this case, the challenges should be simple from a motor perspective, as they have a lot of emotional involvement linked to subjective risk.

The combination of these two approaches helps students develop social and emotional skills, and improves effective communication, conflict resolution, and empathy. In this way, a more effective and enriching experience is achieved in PE sessions.

### 4.3. Health-Based PE Model and Attitudinal Style

The health-based PE model aims to enhance overall well-being through exercise and related activities. It includes recreational and competitive pursuits, along with activities focused on improving basic motor skills [40,41]. Reference [42] specifies the key features of the model, emphasizing PE’s connection with the social reality of the immediate context. It underscores the importance of nurturing interpersonal interactions in the classroom to foster student engagement in current and future physical activity practices. Additionally, there is an emphasis on gradually increasing time spent on health promotion in educational settings.

According to [43], the aim is to cultivate students who are regular practitioners of physical activity and well-informed about maintaining and improving their health. This involves integrating proposals to enhance student autonomy and foster positive experiences within the Adventure Sports (AS) framework [2], characterized by self-regulation and formative assessment [20]. This integration is expected to encourage students’ commitment to physical activity beyond school hours, improve students’ autonomy, develop interpersonal relationships, and enhance health promotion in school environments.

The hybridization of AS and the health-based PE model, coupled with the application of self-regulation-based strategies, enables the PE teacher to sequence the development of healthy habits and physical activity throughout each developmental stage. Some summarized are provided below. Note that these examples are based on the Spanish educational system:

(1) Treatment of the physical condition block: In the 1st and 2nd grades (11–13 years old), the approach begins with the proposals that students start managing their running pace through time control. This includes adapting this activity to the aquatic environment [19], where students learn to swim at a specific pace. Additionally, students participate in an athletics competition. Here, they manage and self-regulate their own learning based on simple templates featuring exercise sequences. Students also autonomously design a brief warm-up, using materials provided by the teacher.

In 3rd grade (14–15 years old), the focus shifts to work regarding the development of strength and endurance routines [20]. Here, students utilize a digital journal to record their endurance and strength workouts, aiming to transfer these skills to their daily lives. All these proposals favor addressing one of the key ideas of the health-based PE model in the classroom, which aims to promote student involvement in the practice of physical activity in the present and future [43]. If the student is able to self-regulate their practice, finding positive experiences in this process, it is more likely that they will engage in physical activity in their leisure time in the future.

Beyond this sequence, and related to another key idea of the health-based PE model—increasing the time dedicated to health promotion in educational centers—proposals at the school level can be implemented.

(2) The Center’s Nutrition and Health Congress can provide an example. On this occasion, students, using a list of topics provided by the teacher, work in pairs or groups of three to create a written script of 4000 words based on TEDx talks. They then deliver a brief oral presentation and collectively organize a Nutrition Congress at the school, attended by students from lower grades (11–16 years old). The ‘Physical Teacher project’ is another example, where students take on the roles of personal trainers and instructors, presenting some classes, such as Total Workout, Body Combat, Yoga, and Body Balance. In this proposal, they organize, structure, and conduct classes at the school once they have acquired the necessary knowledge in PE classes.

In all these proposals, the application of a formative and shared assessment plays a crucial role, especially as it pertains to health education. For instance, in the strength-resistance work employed within the AS, the importance of daily self-assessment and self-regulated learning, and the development of personal responsibility, are highlighted as enhancing motivation, planning, and student engagement in daily physical activities [20]. This can align with the health-based PE model, as a formative assessment using the AS considers aspects beyond physical skills to contribute to the overall well-being of students. It promotes active and healthy lifestyle habits through continuous assessment and the development of personal and social skills.

The hybridization of both models offers an exceptional opportunity to facilitate the transferal of content acquired in the classroom to real-life settings and to encourage the adoption of healthy habits among students.

### 4.4. Adventure Educational Model and Attitudinal Style

The Adventure Educational Model (AEM) represents a distinct type of PE curriculum. It is based on involving students in group tasks and exposing them to various physical activities that are not commonly found in traditional PE settings [4]. Integrated adventure-based learning programs are grounded in five key concepts: challenge, cooperation, risk, trust, and problem-solving [44]. Additionally, reference [45] adds experiential learning and contact with nature to these key elements. The rationale behind this model lies in the creation of experiences, outings, excursions, outdoor expeditions, and contact with nature, providing students with practical opportunities for both group and individual development [46]. Reference [47] outlines the key characteristics of this model, which include the following: (1) engaging in unconventional activities in spaces or facilities unfamiliar to or rarely used by students, (2) working with small groups, (3) incorporating activities that engage students both physically and cognitively, (4) fostering problem-solving skills within the activities, and (5) implementing a minimum of four sessions, aiming to alternate between work outside and inside the school premises.

The application of the AEM in the classroom has demonstrated benefits in psychological and academic variables [48], as well as improvements in self-concept and physical well-being [49]. All these outcomes align with the model’s intentions, to which [45] adds competency-based learning and the holistic development of students. These aspirations are in clear harmony with what is reflected in the AS, which has an explicit focus on competency development.

In this regard, a proposal developed within the framework of the AS that can be implemented in conjunction with the AEM is exemplified. It aims to develop a more competency-based and interdisciplinary focus when overcoming obstacles [22]. Thus, in PE sessions, students are taught the use of certain concepts, such as a movable pulley or friction, while also learning to tie knots and the necessary procedures for tensioning a rope to overcome obstacles. Subsequently, in physics and chemistry, a series of activities are proposed to gather real data on rope tension.

On the other hand, breaking the psychological boundaries of students through intentional bodily activities based on simple tasks that everyone can achieve is a key component of the AEM to promote motivational aspects and perceived self-efficacy. In this regard, in the experiential and self-awareness phase proposed by [45], activities based on a sequential organization of attitudes can be included. Here, students take on various roles, using their own characteristics and also those of their peers. This point is key in breaking the psychological barriers of students. The subjective risk perceived by the students is high, even though a task may be simple from a motor perspective. This restricts their willingness to engage in the task.

This sequence will facilitate the transition from activities performed individually or in pairs to tasks in small groups, where individual responsibility is added, as cited by [45]. It is worth noting that this sequence can be altered based on the evolution and characteristics of the class group. At this point, a formative assessment could be added to promote students’ reflective process through self-evaluation mechanisms. In the initial phase, a personal journal could be used. In this journal, students would reflect on the sensations experienced in the session, their initial fears, or challenges overcome in the classroom. Furthermore, it delves into the relationships that emerged with other classmates, the assistance provided, or any improvement in interpersonal relationships. Subsequently, various assessment tools designed within the framework of the AS could be used, where group members evaluate their peers’ performance during the proposed activities.

However, the practical phase of ‘challenge activities and problems with minor modifications of the real situation’, as proposed by [45], could be enhanced by adding group challenges. Here, the participation of all the students will be necessary to successfully overcome the challenges. At this point, if applying the AS, competitive activities will be avoided. This is in order to provide a collaborative and cooperative nature in which everyone participates and contributes to the benefit of the group. In this sense, taking the activity proposed by [45], ‘sum of steps’, as a reference, students on the wall bars must make specific movements and grips in a sequential order so that everyone can perform all the steps. To do this, they must take into account the characteristics of their peers, as well as help, advise, and give feedback at all times. In these group challenges, reconverted into final assemblies using a collaborative approach, students can incorporate all the previously learned aspects of the lesson, dialogues, and even the use of other materials.

This final assembly, along with others carried out throughout the course, will be crucial in fostering greater cohesion within the group, enabling more successful outdoor activities that allow for contact with nature—for instance, a week-long end-of-course trip to the Pyrenees. During these trips, various activities can be carried out in the natural environment (climbing, rappelling, canyoning, caving, etc.). These activities may have been previously addressed in the classroom under the simulated situations proposed by the AEM. Certainly, individual and group responsibility, overcoming fears, mutual assistance among peers, and a sense of achievement will emerge at this point if the work completed throughout the course has been appropriate.

The hybridization of both models occurs naturally, as they share the same philosophy of work and intentionality, understanding affective–emotional work as a determining aspect that must be addressed before the motor work itself. Their joint application strengthens the implementation of the AEM in the classroom and contributes more directly to the competency-based work and holistic development of students.

## 5. Key Elements for Hybridizing the AS

Using AS as a reference MP in its hybridization with other models enhances the treatment of different content in the classroom. Examples of this include the application of CL (e.g., country), TGfU (e.g., team sports), the AEM model (e.g., obstacle overcoming), or the health-based PE model (e.g., physical condition). However, when implementing these hybridizations, it is crucial for the PE teacher to consider various key components of AS to maintain its essence.

As highlighted in the article, in AS, group achievement is essential. Therefore, it is necessary to initially foster a positive working climate in the classroom. An effective strategy is to start with activities that allow students to achieve individual successes, thereby strengthening their confidence and helping them to effectively contribute to group challenges (such as final assemblies). In these activities, interpersonal relationships are decisive, so the PE teacher should pay special attention to sequential organization (groupings) to manage the different learning paces within the class group. 

Another key aspect that is worth highlighting is the need to specify the evaluable portions of the unit at the beginning. These will be the benchmarks by which students will demonstrate what they have learned, and which will be used to implement a formative assessment.

## 6. Conclusions

This study has thoroughly explored the possibilities of the hybridization of the AS with various PMs in the context of PE. Throughout the analysis, it has been demonstrated that the AS can effectively be integrated with other PMs, enhancing teaching and learning in PE.

In each of these hybridizations, the synergy between the AS and other PMs is evident. This highlights how a combination of approaches can enrich PE by addressing cognitive, motor, affective–emotional, social inclusion, and interpersonal relationship aspects simultaneously. This comprehensive approach, supported by the joint application of various models, not only strengthens the acquisition of physical skills but also contributes to the development of positive attitudes, thus promoting competency-based learning and the holistic development of students. 

Additionally, future research should delve into the experiences and opinions of the teachers who implement these hybridized approaches. Their insights can provide valuable feedback for refining and optimizing the integration of the AS with other PMs in PE instruction. By addressing these areas of inquiry, we can continue to advance our understanding of how hybridization can positively influence competency-based learning and holistic student development in the field of PE.

## Figures and Tables

**Table 1:** Summary of selected studies on the hybridization of the AS with other PMs in PE.

Reference	PMs Hybridized	Content	Key Findings
[19]	Health-based PE, Self-regulation and AS	Physical Condition	Proposal regarding swimming at a controlled pace using self-regulation.
[20]	Health-based PE, Self-regulation and AS	Physical Condition	Proposal on how to implement the AS, health-based PE, and the self-regulation model throughout the Secondary stage to address the treatment of physical fitness content.
[21]	CL and AS	Body expression	An approach to country dance through CL and AS.
[22]	AEM and AS	Natural environment	AS, when combined with the AEM, is pursued to foster a more competency-based and interdisciplinary approach within content related to overcoming obstacles.
[23]	TGfU and AS	Sports	Formative assessment and its relationship with the AS. First practical application in a sports unit.
[24]	CL and AS	Physical Condition	Cooperative physical challenges of an emotional nature within the framework of AS.
[25]	CL and AS	Natural environment	The AS and content in the natural environment: a proposal on how to overcome obstacles and complete rope work using the AS and CL.

## Data Availability

For this narrative review article, as it does not involve the generation of new data or a specific quantitative analysis, no new data were created. The study is based on a comprehensive review of existing literature, and the results and conclusions are derived from the analysis and synthesis of previously published information.
